# The Shared Safety Net Action Plan (SSNAP): a co-designed intervention to reduce delays in cancer diagnosis

**DOI:** 10.3399/BJGP.2021.0476

**Published:** 2022-04-05

**Authors:** Jane Heyhoe, Caroline Reynolds, Remi Bec, Daniel Wolstenholme, Cheryl Grindell, Gemma Louch, Rebecca Lawton

**Affiliations:** Bradford Institute for Health Research, Bradford.; Medical Examiner Office, Bradford Teaching Hospitals NHS Foundation Trust, Bradford.; Lab4Living, Sheffield Hallam University, Sheffield.; Royal College of Obstetricians and Gynaecologists, London.; School of Health and Related Research, University of Sheffield, Sheffield.; Bradford Institute for Health Research, Bradford.; Psychology of Healthcare, School of Psychology, University of Leeds, Leeds; Bradford Institute for Health Research, Bradford.

**Keywords:** cancer, co-design, diagnosis, primary health care, safety-netting, focus groups

## Abstract

**Background:**

Safety netting in primary care may help diagnose cancer earlier, but it is unclear what the format and content of an acceptable safety-netting intervention would be. This project aimed to co-design a safety-netting intervention with and for primary care patients and staff.

**Aim:**

This work sought to address how a safety-netting intervention would be implemented in practice; and, if and how a safety-netting intervention would be acceptable to all stakeholders.

**Design and setting:**

Patient representatives, GPs, and nurse practitioners were invited to a series of co-design workshops. Patients who had and had not received a diagnosis of cancer and primary care practices took part in separate focus groups.

**Method:**

Three workshops using creative co-design processes developed the format and content of the intervention prototype. The COM-B Framework underpinned five focus groups to establish views on capability, opportunity, and motivation to use the intervention to assist with prototype refinement.

**Results:**

Workshops and focus groups suggested the intervention format and content should incorporate visual and written communication specifying clear timelines for monitoring symptoms and when to present back; be available in paper and electronic forms linked to existing computer systems; and be able to be delivered within a 10-minute consultation. Intervention use themes included ‘building confidence through partnership’, ‘using familiar and current procedures and systems’, and ‘seeing value’.

**Conclusion:**

The Shared Safety Net Action Plan (SSNAP) — a safety-netting intervention to assist the timely diagnosis of cancer in primary care, was successfully co-designed with and for patients and primary care staff.

## INTRODUCTION

Diagnostic delay for cancer can occur at different stages during the diagnostic process.^1^^,^^2^ The period from when a patient presents to primary care with symptoms until referral is one of the points when missed opportunities may occur. For example, a national primary care audit found 12.5% of patients experienced a delay of >60 days from first presenting with relevant symptoms until referral for suspected cancer,^3^ and 41% of 3298 UK patients whose cancer was diagnosed as an emergency had ≥3 GP consultations in the previous weeks and months.^4^

Cancer does not always present with ‘red flag’ symptoms, and vague or atypical symptoms can make diagnosis difficult.^5^^,^^6^ Literature and NHS policy suggests that involving patients in ‘safety netting’ could support the early detection of cancer^7^^,^^8^ and finding ways to do this is now a priority.^9^

The concept of safety netting in primary care originated from three questions that Neighbour^10^ posits should be considered when dealing with uncertainty in primary care. These are:

- If I’m right, what do I expect to happen?
- How will I know if I’m wrong?
- What would I do then?

While work by Almond and colleagues^11^ identified safety-netting advice that clinicians can impart to patients during the consultation (the doctor and patient talking openly about there being uncertainty about what is causing the patient’s symptoms, when they should expect symptoms to get better, what symptoms they need to continue to look out for, and when to come back to see the doctor again), literature highlights that there is variation in how safety netting is applied and a lack of consensus about what and who it should involve.^7^^,^^12^^–^17 There is also increasing recognition of the need to explore the role that patients may have in assisting in diagnosis^17^^,^^18^ and how patient engagement in diagnosis might be achieved.^19^

A review completed by the study team identified no interventions that involved patients in safety netting following an initial presentation to primary care and before a referral or diagnosis is obtained, and proposed a logic model for interventions of this kind.^20^ Previous qualitative research with patients and stakeholders in one UK region identified factors that hinder or encourage patients to assist with diagnosis in primary care and also explored possible interventions that might facilitate patient involvement in achieving a faster cancer diagnosis in a primary care setting.^21^ This study builds on this previous review and qualitative research findings.^20^^,^^21^ In previous work, patients and health professionals prioritised the need for an intervention to 1) provide patients with a symptom review prompt at the end of their first consultation; 2) provide information to enhance patients’ understanding of the decision to re-attend; and 3) support appropriate re-attendance at the practice. This approach supports recent literature that recommends the need to establish clear actions and practices for safety netting in primary care,^7^^,^^12^ and which suggests that safety netting should involve aspects that are psychological (for example, legitimising repeat visits), cognitive (for example, checking patient’s understanding), and informative (for example, discussing concerning symptoms or signs to look out for).^15^ Working collaboratively, the Yorkshire Quality and Safety Research Group (psychologists and health services researchers) and the Translating Knowledge into Action theme of the National Institute for Health Research Collaboration for Leadership in Applied Health Research and Care Yorkshire and Humber (a team of clinical and design researchers) aimed to co-design the format and content of a safety-netting intervention that promoted greater involvement of patients to support the timely diagnosis of cancer in primary care.

**Table:** How this fits in

Finding ways to diagnose cancer earlier is now a priority. Safety netting may assist the earlier detection of cancer, but it is unclear what the format and content of an acceptable safety-netting intervention for use in primary care would involve. Using creative co-design processes and a phase of stakeholder input and feedback, knowledge was gained about the components considered essential for a safety-netting intervention to work in practice that can be used with patients following an initial presentation to primary care and before a referral or diagnosis is obtained. In particular, patients and primary care staff identified important principles for a safety-netting intervention, such as encouraging staff and patients to discuss uncertainty about diagnosis, providing patients with a symptom review prompt post-consultation, and providing patients with a plan for returning to primary care if necessary. The safety-netting intervention, co-designed with and for patients and primary care staff, can now be examined to assess whether it is feasible and potentially effective in practice.

## METHOD

To address the aim, Medical Research Council guidance on development of a complex intervention^22^ was utilised as well as contemporary thinking in user-centred and creative co-design.^23^^,^^24^ Primary care stakeholders took part in the process to co-produce solutions to fit with current lifestyle and practice. In particular, the purpose of the co-design workshops was to develop the format and content of the safety-netting intervention prototype. Focus groups provided feedback on the prototype to assist with further refinement.

Intervention components prioritised by stakeholders during previous work^21^ and new knowledge gained during this current work was discussed by the research team during project management group meetings. This cycle of interlinked stakeholder input and feedback continued until a final prototype was agreed. Processes and outputs are described below.

### Recruitment

Regional patient representatives and healthcare professionals were invited to attend a series of workshops to develop the format and content of the safety-netting intervention prototype. Nine participants attended Workshop 1. Participants included seven patient representatives and two primary care staff (one GP and one nurse practitioner). Eleven participants attended Workshop 2. Participants included seven patient representatives and four primary care staff (three GPs and one nurse practitioner). Seven participants attended workshop 3. Participants included five patient representatives and two primary care staff (two GPs). While patient representatives, unless unable to attend, consisted of the same individuals across the three workshops, primary care staff varied due to availability.

Focus group participants were recruited from one region in northern England. Patients were recruited via local cancer support groups, general local community groups, health research groups, and cancer forums. Both patients who had received a diagnosis of cancer and patients who had not received a diagnosis of cancer were included and a separate focus group was held for each patient group. Primary care practices were recruited via circulation of study information via the clinical commissioning group.

The inclusion criterion was that practice staff had experience of being involved in the diagnosis of cancer in primary care. A focus group was held for staff at each practice. The focus group at practice 1 included two GPs, and one GP from practice 2 and practice 3 took part in the focus group. Focus group participant characteristics are illustrated in Table 1.

**Table 1.** Focus group participant characteristics

**Participant characteristics**	**Focus groups,** ***n***	

	**Patients**	**Primary care staff**
Participants	10	21

Age range, years	39–73	29–60

**Sex**		
Male	4	3
Female	6	16
Not stated	0	2

Clinical experience range (if applicable), years	—	1–36

Patients diagnosed with cancer	5	—

Patients not diagnosed with cancer	5	—

GP	—	4

Practice nurse	—	3

Healthcare assistant	—	1

Practice manager	—	3

Office manager	—	1

Administrative staff	—	8

Role not stated	—	1

### The intervention development process

This section describes the processes utilised in the intervention development process and related outputs.

### Workshops 1 and 2

Guided by previous research,^20^^,^^21^ two workshops were planned to develop the format and content of the intervention.

#### Workshop 1: Task 1

Involved using a visual representation of the concept of safety netting (see Supplementary Figure S1 for visual used in Workshop 1 to define what ‘safety netting’ means) to introduce the project to the participants and ensure all stakeholders had a shared understanding of safety netting. Positive and negative experiences of primary care consultations were also explored. To facilitate a discussion around a shared understanding of safety netting within a primary care consultation and context, Neighbour’s^10^ original definition of safety netting and the three questions Neighbour suggests should be considered when dealing with uncertainty in primary care were used.

#### Task 2

Involved small group work to create a set of personas (fictional characters created by stakeholders) that represented both staff and patients (for example, paternalistic staff and worried well or stoic patient).

#### Task 3

Involved each group creating a ‘mood board’ around their personas as a way to stimulate stakeholders’ thinking about different behaviour and attitudes towards self-management and safety netting from both a provider and patient perspective.

#### Workshop 2

Following a presentation to recap on knowledge gained from Workshop 1, Task 1 involved the stakeholders considering the physical context of the GP surgery where the safety-netting intervention would take place. Using the design ‘brief’, *‘Design an intervention to assist the timely follow-up and review of patients with inconclusive diagnosis (safety-netting)’*, and a GP and patient persona created in the previous workshop, stakeholders were divided into two groups (each group consisting of patient representatives and primary care staff) and asked to consider how key questions for each character might be addressed in an intervention, for example:

*‘How do you get John to come back if his cough gets worse? How do you get Dr Lahari to develop a shared plan with Denise?’*

#### Output

The core team met to reflect on the outputs of the workshops and created version 1 of the prototype, named the ‘Safety Netting Shared Plan’.

### Focus groups: prototype v1

Four focus groups, lasting 42 to 81 minutes, took place after Workshop 2 to gain feedback about the format and content of prototype v1 for further refinement, and to explore what would need to happen to support implementation of the tool (prototype v1 with two patient and two staff focus groups). Participants were provided with a written illustration of prototype v1 and the researchers conducted a short roleplay that presented the main components of the intervention. The focus group topic guide (see Supplementary Box S1 for focus groups topic guide) was underpinned by the COM-B Framework.^25^ This theoretical framework allowed the capability, opportunity, and motivation of both patients and staff to be assessed to use the intervention and to develop an understanding of their response in terms of contextual and behavioural factors.

Participants were also asked to consider whether any modifications were required. In addition, primary care practice staff were asked to consider whether they felt the intervention mechanisms and components proposed were achievable or problematic in practice. All focus groups were audiorecorded for analysis and transcribed verbatim.

#### Workshop 3

The aim of the final workshop was to present key feedback from the first four focus groups and use this feedback to further refine the content and format of prototype v1. Stakeholders were divided into two groups (each group consisting of patient representatives and primary care staff), presented focus group feedback about key components of prototype v1, and asked to consider the feedback and provide solutions for further refinement.

#### Output

The core team met to reflect on the outputs of the workshop and created version 2 of the prototype, prototype v2, which was taken to the final focus group with practice staff.

### Focus group: prototype v2

The final focus group lasted 43 minutes, followed the same design and method as the focus groups described previously, and explored with primary care staff the format and content of prototype v2.

### Analysis

#### Workshops

Data were analysed after the workshops, which led to the identification of key issues and considerations. The workshops’ analysis directly informed the activities and content of the subsequent workshop.

#### Focus groups

Analysis was completed by hand and Excel software was used to arrange data. Thematic analysis was used to organise data.^26^ First, transcripts were coded. For analytical rigour and to control for subjectivity, 40% of the transcripts were independently double coded by another researcher. This allowed for the two researchers to cross-check and refine codes. Key concepts were then drawn from the codes to develop themes underpinned by the COM-B Framework^25^ and were discussed during meetings. The final themes were agreed through consensus.^27^

## RESULTS

This section presents the results from the three workshops and the five focus groups.

### Workshops 1 and 2

The first two workshops generated a first working prototype. Key factors for considerations arose from Workshop 1 with regards to the primary care consultation. These included the importance of relationship building between patient and GP (for example, communication skills, and how a negative experience could influence whether a patient would come back), information sharing and explanations (for example, regarding potential diagnosis), true shared decision making (feeling part of the process), and development of a management plan. Workshop 2 identified specifics related to the look, feel, content, and practicalities of the potential intervention.

The data showed that stakeholders would like visual as well as written forms of communication, including key timelines (how long to monitor symptoms and when to present back), that could be delivered within a 10-minute consultation. The intervention needed to help patients recognise the need to come back for a review (for example, a traffic light system) and that as well as a paper form, the intervention should be available in electronic form linked to existing computer systems (for example, SystmOne).

### Output: prototype v1

**Figure 1.**
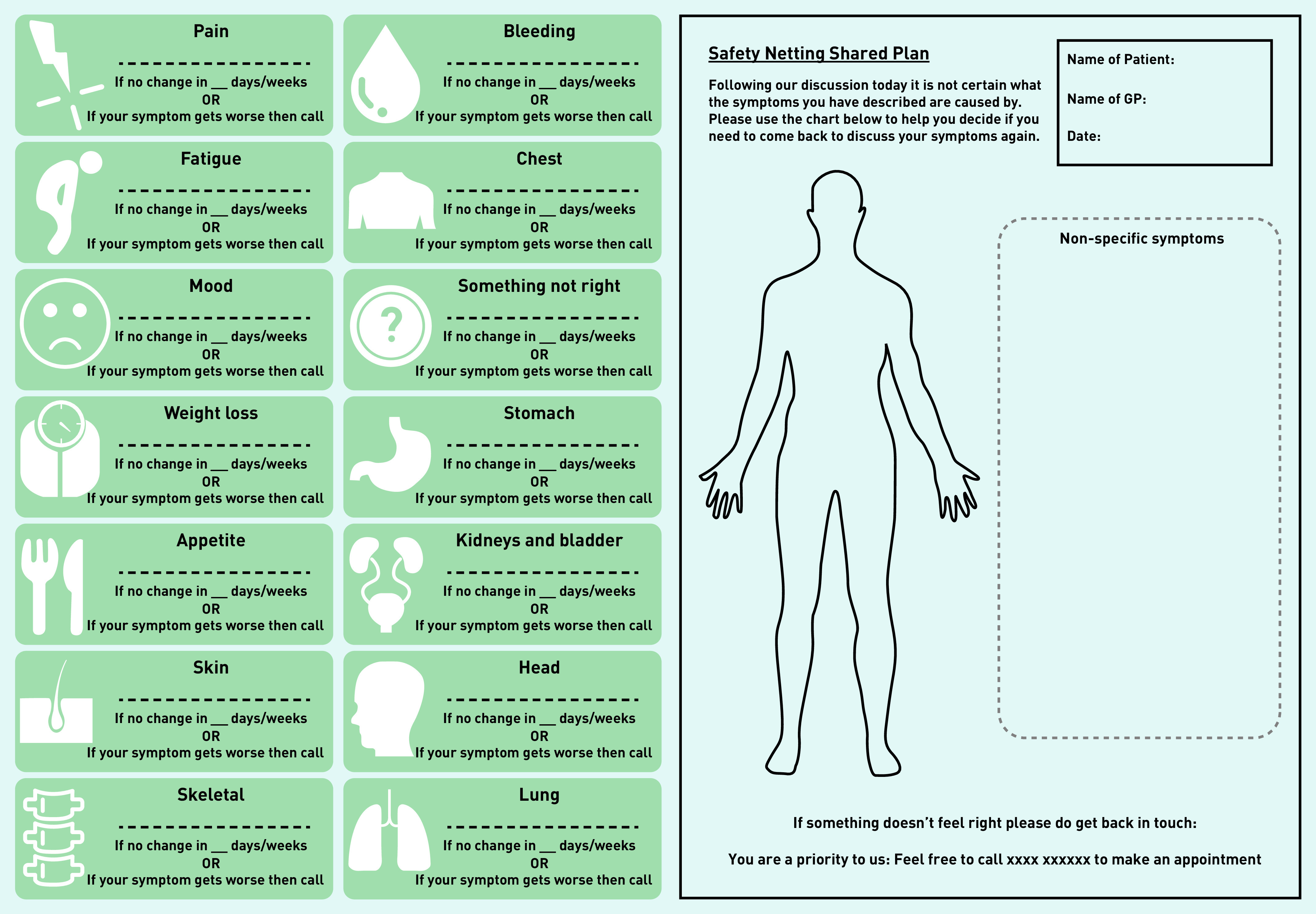
*Prototype v1.*

The development of the first prototype started with a visual representation of the ideas generated from Workshops 1 and 2 through rough sketching (see Supplementary Figure S2 for initial safety-netting intervention idea). Once the basic concept of a body chart indicating patient symptoms to be monitored was agreed on by the core team the design researcher developed this further. The final iteration of prototype v1 included a printed body chart and anatomical symptom icons that could potentially be physical stickers used on a paper version, or icons embedded within an online template and used by the patient and GP together to develop a safety-netting plan to help keep track and report those symptoms should they persist, change, or get worse within an agreed time scale (Figure 1). Consideration was also given to the use of a tab within an electronic system that could generate a text prompt that could be sent to the patient at a specified time after the consultation. It was proposed that the wording could be: *‘This is a reminder from your GP to check your safety-netting prescription/shared plan and to get back in touch if needed. Tel: (insert number).’*

**Figure 2.**
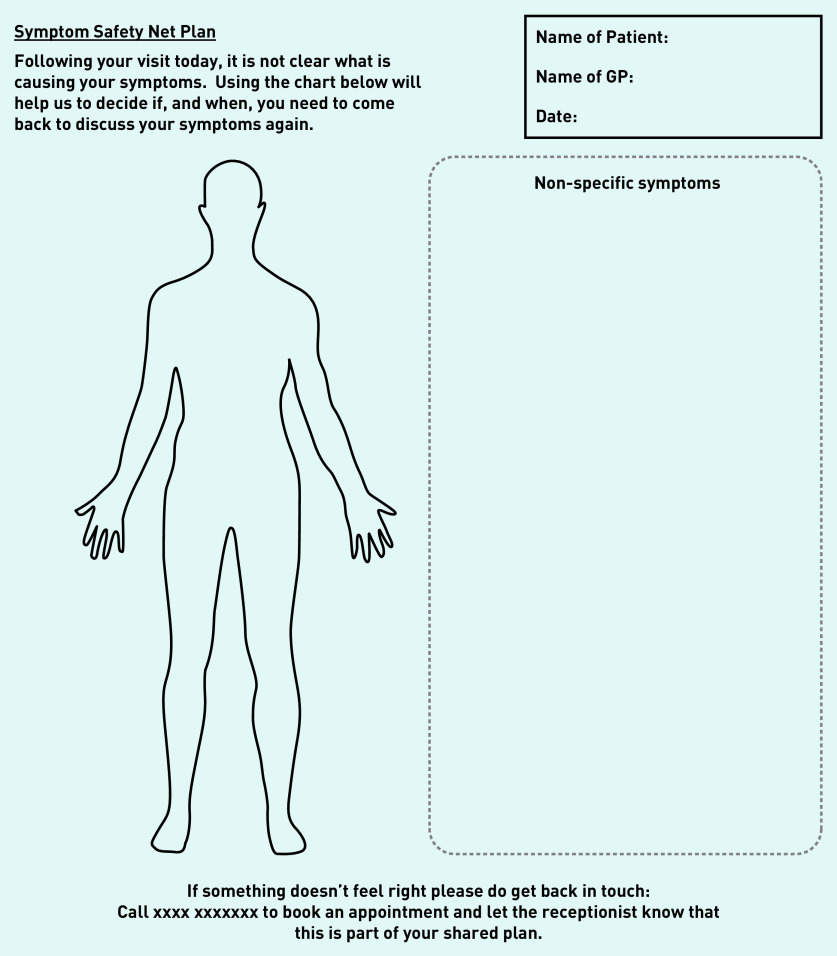
*Prototype v2 – Revised plan.*

Figure 1 shows the first prototype of the body chart and icons based on ideas generated in Workshop 2. These icons along with the body chart and text prompt wording were presented to participants in the four focus groups, which led to further prototype iterations (Figure 2).

### Focus groups: prototype v1

Themes concerning the use of the safety-netting intervention were identified from patient and staff discussions. ‘Building confidence through partnership’ and the importance of ‘a practical and clear plan’ emerged as being key to capability to use the safety-netting intervention. Consideration about the opportunity to use the safety-netting intervention focused on issues around ‘using familiar and current procedures and systems’ and ‘patient factors’. Motivation to use the safety-netting intervention was discussed in terms of ‘seeing value’, ‘efficient and cost effective care’, ‘earlier diagnosis’, ‘safe care’, and ‘confidence in the benefit of the intervention’. Theme explanations and exemplar quotes are provided in Supplementary Table S1 (focus groups pertaining to prototype v1 COM-B themes with illustrative quotes). Feedback from staff also identified key themes around the acceptability and feasibility of the safety-netting intervention in an organisational context.

The themes, ‘requires clear strategies’ and ‘system implications’, are presented with exemplar quotes in Table 2. These provided early directions for implementing the prototype into primary care practice and how it might be part of a wider service delivery.

**Table 2.** Focus group organisational themes with illustrative quotes

**Themes**	**Illustrative quotes**
**Requires clear strategies**	
Potential problems and work flow issues were discussed when considering mechanisms. An important aspect was that staff felt that there needed to be clarity about what would happen once a text prompt had been sent to the patient. In particular, there was concern about what should be done if patients did not re-attend. It was suggested that enabling patients to reply to the text prompt would avoid this scenario.	*‘For the prompt I think you would need a good text messaging service so we have got something called Mjog which allows patients to text back so maybe if when they have got a prompt they could maybe text back you know, 1. No symptoms any more so then you still have that feedback if it has completely resolved itself and it hasn’t just gone out into the ether and you haven’t got any feedback.’* (Practice 2)

**System implications**	
Staff referred to mechanisms already in place when considering whether the intervention would work. It was felt that current mechanisms would support the intervention being embedded into existing electronic templates, providing patient copies, the generation of a text message prompt, and patients being able to reply to the text prompt. However, staff also highlighted potential technical difficulties if practices had different IT systems, necessitating the need for different versions of the intervention. There was discussion about how IT may act as a barrier to the uptake of the intervention and that issues around IT were a major consideration for embedding new practices in primary care. Staff emphasised the importance of working with system developers to successfully implement the intervention in practice.	*‘We frequently give patients regular information don’t we?’* (Practice 1) *‘… it is just time and IT isn’t it? And again what might work well in SystmOne which where a system on practice might not work well in an EMIS practice. I think you would have to have several different versions based on the clinical system and the ability and again text messaging services it depends what practices have signed up for and are willing to pay for as to how it would work.’* (Practice 2)

### Modifications

A number of suggestions for intervention modification were made including:

- alterations and additions to wording, such as adding the GP’s name to the text prompt for authenticity and to encourage use;
- revising the icons to make symptoms, timescales, and actions clearer;
- removing the term ‘prescription’ from the text prompt to avoid confusion with the current meaning; and
- changes to the mechanisms of the tool, such as the method of delivery and responding to the text prompt were also proposed.

### Workshop 3

The feedback gathered during the first four focus groups allowed insights to be obtained to improve the content of prototype v1 and more importantly to foresee how it can be integrated into clinical practice. Based on focus group feedback, it was explored further in Workshop 3 how the prototype would work in clinical practice from a stakeholders’ perspective. The content and format of the shared plan and prompt as well as the various mechanisms (for example, documentation of agreement, and recording information) were also considered.

### Shared plan: content and format

When considering using the intervention in practice, it was agreed clear strategies are required to ensure support (that is, guidelines) to assist with decisions and the management of follow-up appointments. Stakeholders reiterated the importance of the shared plan, including the length of time to monitor symptoms and instructions about what to do if symptoms become worse. It was felt that it should facilitate an enhanced understanding that uncertainty is part of diagnosis. While it was felt that it should support discussion for making a future face-to-face appointment, stakeholders agreed that the healthcare professional should decide on how urgent an appointment should be. The inclusion of free text was also viewed as essential for capturing information that might assist diagnosis. Stakeholders also considered whether the free-text section could be used by patients to monitor symptoms and provide more diagnostic information such as length of time, number of times, and severity. Stakeholders suggested further names for the intervention, which included ‘uncertainty management plan’, ‘uncertainty tracker/follow-up plan’, ‘symptom tracker’, ‘symptom monitoring’, ‘symptom safety-net/safety plan’, and ‘managing my care’.

While there was a perception that healthcare professionals liked ‘safety-netting shared plan’, stakeholders generally agreed that the word ‘shared’ was important and should be considered as part of the name.

### Prompt: content and format

Stakeholders liked the idea of the healthcare professional’s name being included in the prompt and suggested adding date of consultation and the practice number to assist with booking an appointment. It was agreed that the message should be consistent for all patients. Stakeholders highlighted specific issues that would need to be considered further, such as: responsibility to respond, how to reply to a prompt (that is, reply to a text message or phone the practice), no reply as an option, prompts sent to carers with prior permission, patients not able to access email and text prompts, whether prompts should be automated, and timings of prompts.

### Mechanisms: agreement, recording, and delivery

Stakeholders suggested that a ‘safety-netting’ tab could be incorporated into an electronic template to ensure inclusion in the patient’s notes, and discussed whether this would be required to document patient agreement and to satisfy legal requirements. It was felt that there should also be a free-text box where more specific information could be recorded about symptoms to assist diagnosis, and potentially a need to record two levels of information collected during the consultation — that which would be more meaningful to staff and be kept in the practice and included in medical notes, and that which would be meaningful for patients and would be shared as part of the plan. It was also felt that there should be an option to amend the plan as and when required.

### Output: prototype v2

Based on the initial focus group feedback and further input from stakeholders during Workshop 3, the prototype was further refined. While the symptom icons remained the same, wording on the plan was revised (Figure 2). Prototype v2 was then taken to a final focus group with practice staff.

### Focus group: prototype v2

Staff feedback mainly supported the Workshop 3 themes described above and are therefore not reiterated here. When considering using the intervention in practice, staff felt that it would require clear strategies and it would be helpful to have support in the form of guidelines to assist with decisions and the management of follow-up appointments:

*‘... there needs to be clear guidance flow diagram for whoever is going to be on the end of the phone really … ’*

(Practice 3)

Staff did discuss the content of the prototype and made suggestions about how it could be further refined. In particular, staff considered the possible effects of referring to ‘uncertainty’ in the safety-netting shared plan. Staff also considered what the intervention should be called with one participant suggesting, *‘symptom shared plan or something* . *’* Differing views about whether the term ‘safety net’ was meaningful were also conveyed. Some staff felt it was appropriate, *‘It’s pretty self-explanatory isn’t it? It’s just a net to catch … ’* while others were unsure, *‘I don’t think so no, if I weren’t a nurse I don’t think I would know, or is that just me?’* (Practice 3)

### Output: prototype v3

**Figure 3.**
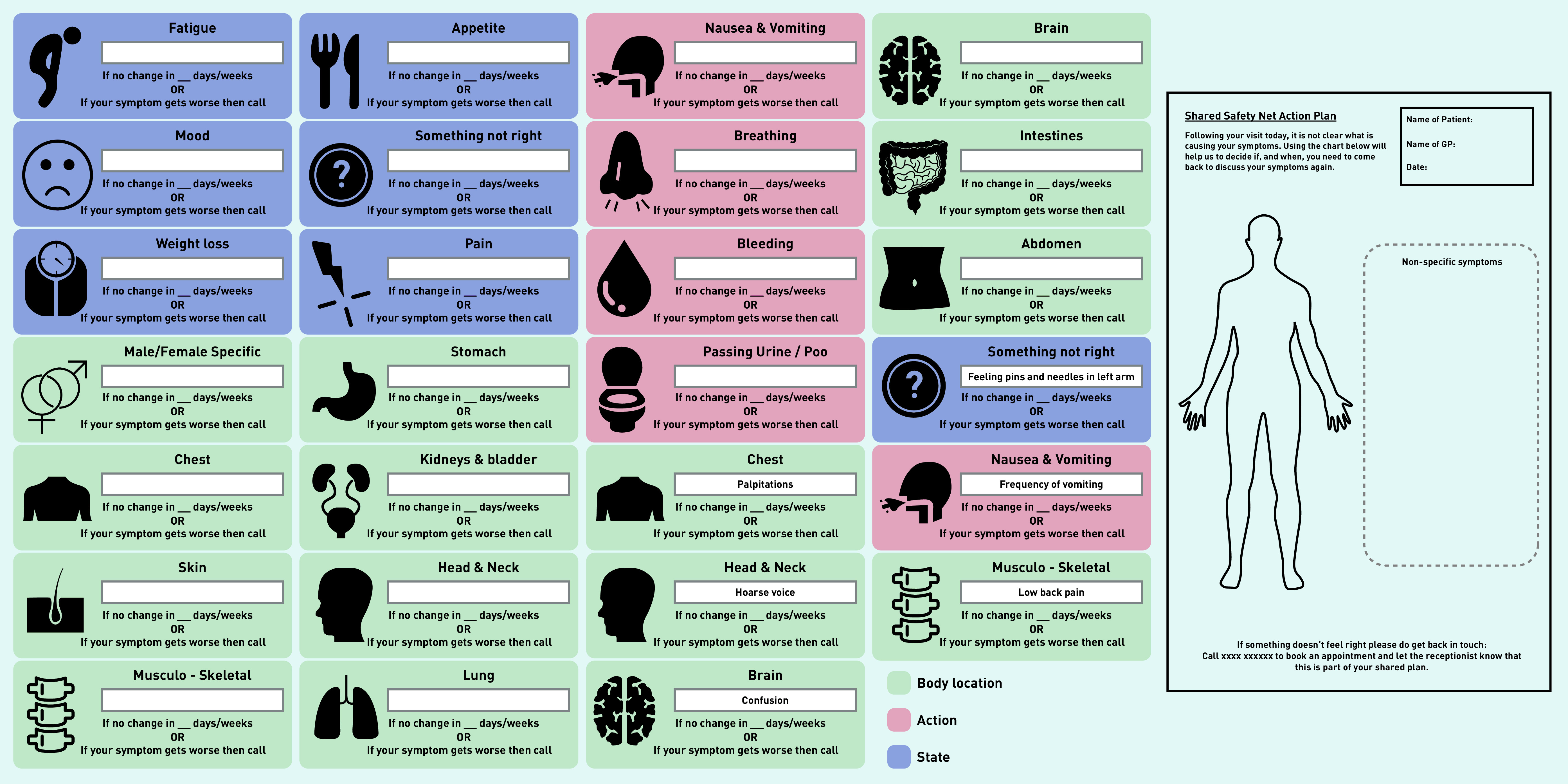
*Prototype v3.*

Following feedback from the final focus group, prototype v3 was developed (Figure 3). This included revising the name of the plan and adding the word ‘Shared’, the addition of colour coding to group symptoms into three categories (‘Body location’, ‘Action’, and ‘State’), and a more defined free-text box within each symptom icon to ensure clarity about the symptom/s that need to be monitored and how long the symptoms should be monitored for. To support this, a white box was added to allow the clinician to adapt the form in line with the agreed plan. Duplicate symptom icons with example text were included to illustrate this. To facilitate understanding about how the paper prototype would be implemented in practice by staff and patients a storyboard was developed (see Supplementary Figure S3 for visual summarising the imagined solution — Shared Safety Net Action Plan [SSNAP]).

### Refinement of symptom icons

The symptom icons represented in prototype v3 were sense checked using the signs and symptoms of cancer^28^ guidelines and by two GP collaborators with research and clinical expertise specific to diagnosing cancer and vague/atypical symptoms. This was to ensure the face validity and clinical relevance of the symptoms, the visual representations of the symptoms, and the category/colour of symptoms as body location, action, or state.

The complete list of refinements made throughout this process of icon refinement can be found in Supplementary Box S2. The refined symptoms icons are included in Supplementary Figure S4.

## DISCUSSION

### Summary

A safety-netting intervention to assist the timely diagnosis of cancer in primary care following an initial presentation to primary care and before a referral or diagnosis is obtained, was successfully developed with and for stakeholders using an approach that incorporated creative co-design.^23^^,^^24^ Knowledge was gained about the principles and components considered necessary for the intervention to work in practice and to be acceptable to all stakeholders, and resulted in an agreed format and content for the final prototype.

### Strengths and limitations

While literature highlights the challenges of bringing different stakeholders together,^29^ the co-creative process used here allowed individuals to have a voice through the creation of an outcome and in the gathering of feedback, and thus encompassed aspects generated by all stakeholders.^24^

Prototypes can be challenging to create, especially when it involves technology. Bec *et al* 30 argued that there are different elements to take into consideration when prototyping. Having developed a low-cost prototype (that is, paper) first was a way to explore the viability of an idea and gain insights to develop it further with minimum resources. In this respect, having a designer within the team becomes useful since they have received training to develop solutions using a combination of 2D and 3D prototypes to communicate an idea. There are numerous techniques designers can use to creatively develop prototypes (that is, using storyboards, developing cardboard mock-ups, creating 3D printed models, and adapting existing technology) within the given constraints (for example, time and budget). The designer’s role in this research allowed the team to communicate the imagined concept effectively through developing visuals (mainly 2D in this case). This supported stakeholders to make the leap between a fictional concept and how this could work in practice.

Due to time commitments, workshops had inconsistent numbers and there were more patient representatives than practice staff. However, three of the five focus groups were with staff, in which all practice staff (clinicians and administrative) were represented, and provided reassurance that use in a practice context was carefully considered and incorporated. While this included co-designing a safety-netting intervention that could be delivered within a 10-minute consultation, the ability of clinicians to use the SSNAP within this timeframe and whether this creates any additional burden now needs to be assessed in practice. Similarly, in order to robustly assess the acceptability of the format and content of the SSNAP, there is a need for testing on a wider scale.

### Comparison with existing literature

Research recommends using clear and robust action plans as part of safety netting in primary care,^9^^,^^13^ yet previous literature highlights a lack of consensus about what should be involved.^7^^,^^12^^–^17 The research team’s^20^^,^^21^ and other recent work^15^^,^^16^^,^^31^^,^^32^ has established the need for strategies to facilitate patient involvement in achieving a faster cancer diagnosis in primary care. In previous research,^20^^,^^21^ patients and health professionals prioritised the need for a safety-netting intervention to provide patients with a symptom review prompt at the end of their first consultation, provide information to enhance patients’ understanding of the decision to re-attend, and support appropriate re-attendance at the practice.

In the SSNAP, following discussion with a clinician in a consultation, symptoms are discussed and illustrated on a body diagram or in ‘non-specific’ symptom charts, a timescale for monitoring symptoms is agreed with the patient and illustrated on charts, this is discussed and agreed between the patient and clinician, the plan is then saved and the patient is given a copy. The patient could then be prompted at a later specified time to review their SSNAP and get back in touch if needed. This work makes a unique contribution by developing an intervention with key components for effective safety netting and a clear process for what should be involved.

### Implications for research and practice

First, small-scale exploratory work is necessary to assess how and why the SSNAP is implemented into primary care consultations and its impact at the practice level. It is also important to explore what further refinement to the SSNAP is required to support the use and implementation of the tool in a primary care consultation and at practice level, and whether there is a need to develop a technical version and what form this may take. In the long term, future research should examine how the SSNAP might be evaluated at scale to demonstrate an impact on the timely diagnosis of cancer and patient outcomes.

To the authors’ knowledge, this is the first safety-netting intervention promoting greater involvement of patients to assist the earlier diagnosis of cancer in primary care that has been co-designed with and for patients and primary care staff. This intervention — the SSNAP — supports important principles for safety netting. It encourages staff and patients to discuss uncertainty about diagnosis, provides patients with a symptom review prompt at the end of their consultation, and a plan for returning to primary care if necessary. The SSNAP represents a way to more routinely implement this in practice in a way that supports the patient to be clear about what they are being asked to do and which gives them permission to re-consult within a specified time frame. While the authors’ previous research tells us there is a need for this intervention and the SSNAP is acceptable to both patients and staff in this study, it is not yet known whether it is feasible and potentially effective in practice or whether it can be evaluated at scale.
